# Optic Neuritis in Typhoid Fever

**Published:** 1897-06-19

**Authors:** 


### OPTIC NEURITIS IN TYPHOID FEVER.

In those doubtful cases which occur sometimes in which it is a matter of extreme difficulty to arrive at a diagnosis between meningitis and typhoid fever, many of us have long pinned our faith on the presence of double optic neuritis, believing that the existence of this condition might fairly be taken as indicating the probability of the disease being meningitis.

Much time and labour have often been expended in the effort to catch a view of the disk in the wandering eye of an unconscious child, with the hope of elucidating the nature of the illness. It is well, then, that we should draw attention to an observation of Mr. Braine-Hartnell, which, however, only confirms what had already been stated by Gowers and Taylor, viz., that double optic neuritis may be met with in typhoid fever without anything being found in the brain to account for it.

